# Individual differences in brain structure underpin empathizing–systemizing cognitive styles in male adults

**DOI:** 10.1016/j.neuroimage.2012.03.018

**Published:** 2012-07-16

**Authors:** Meng-Chuan Lai, Michael V. Lombardo, Bhismadev Chakrabarti, Christine Ecker, Susan A. Sadek, Sally J. Wheelwright, Declan G.M. Murphy, John Suckling, Edward T. Bullmore, MRC AIMS Consortium, Simon Baron-Cohen

**Affiliations:** a Autism Research Centre, Department of Psychiatry, University of Cambridge; Douglas House, 18B, Trumpington Road, Cambridge CB2 8AH, UK; b School of Psychology and Clinical Language Sciences, Centre for Integrative Neuroscience and Neurodynamics, University of Reading; Earley Gate, Whiteknights Road, Reading RG6 6AL, UK; c Department of Forensic and Neurodevelopmental Sciences, Institute of Psychiatry, King's College London; PO23, Institute of Psychiatry, De Crespigny Park, Denmark Hill, London SE5 8AF, UK; d Brain Mapping Unit, Department of Psychiatry, University of Cambridge; Herchel Smith Building, Robinson Way, Cambridge CB2 0SZ, UK

**Keywords:** Individual differences, Cognitive style, Empathy, Systemizing, VBM

## Abstract

Individual differences in cognitive style can be characterized along two dimensions: ‘systemizing’ (S, the drive to analyze or build ‘rule-based’ systems) and ‘empathizing’ (E, the drive to identify another's mental state and respond to this with an appropriate emotion). Discrepancies between these two dimensions in one direction (S > E) or the other (E > S) are associated with sex differences in cognition: on average more males show an S > E cognitive style, while on average more females show an E > S profile. The neurobiological basis of these different profiles remains unknown. Since individuals may be typical or atypical for their sex, it is important to move away from the study of sex differences and towards the study of differences in cognitive style. Using structural magnetic resonance imaging we examined how neuroanatomy varies as a function of the discrepancy between E and S in 88 adult males from the general population. Selecting just males allows us to study discrepant E-S profiles in a pure way, unconfounded by other factors related to sex and gender. An increasing S > E profile was associated with increased gray matter volume in cingulate and dorsal medial prefrontal areas which have been implicated in processes related to cognitive control, monitoring, error detection, and probabilistic inference. An increasing E > S profile was associated with larger hypothalamic and ventral basal ganglia regions which have been implicated in neuroendocrine control, motivation and reward. These results suggest an underlying neuroanatomical basis linked to the discrepancy between these two important dimensions of individual differences in cognitive style.

## Highlights

► Empathizing (E)–Systemizing (S) discrepancy differentiates human cognitive styles. ► We investigate the brain structural basis for ‘E–S discrepancy’ in adult males. ► S > E profile is related to larger cingulate and dorsal medial prefrontal structures. ► E > S profile is related to larger hypothalamus and ventral basal ganglia. ► E–S discrepancy is reflected by distinct individual differences in brain structure.

## Introduction

As a species, humans have had to adapt to complex social and physical environments. These demands are thought to have led to the evolution of two core domains of cognition: ‘folk psychology’ and ‘folk physics’ (Wellman and Gelman, 1992). These are proposed to be the innate ‘building blocks’ for intuitively understanding information from the social and non-social worlds. Within this framework we have proposed there are two major dimensions of cognition: empathizing and systemizing (Baron-Cohen, 2002, 2009; Baron-Cohen et al., 2005).

*Empathizing* is defined as the drive to identify another's mental states and to respond to these with an appropriate emotion (Baron-Cohen, 2003). This encompasses two components of empathy: *cognitive* (also called ‘mentalizing’ or ‘theory of mind’), which is the capacity to recognize what someone else thinks or feels, and *affective*, which is the capacity to feel an appropriate emotion in response to someone else's thoughts and feelings (Cox et al., 2011; Shamay-Tsoory, 2011). Empathizing takes the concept of ‘folk psychology’ further as a powerful process for understanding *agentive* events and interactions in our environment.

*Systemizing* is defined as the drive to analyze and construct *rule-based* systems (Baron-Cohen et al., 2003; Wheelwright et al., 2006). When we systemize we try to identify the ‘input–operation–output’ rules that govern and predict how the system behaves. Systems may be mechanical (e.g., a bicycle), natural (e.g., the tides), abstract (e.g., the syntax of language), collectible (e.g., a library catalogue) or even social (e.g., a football team). Systemizing is a powerful process for understanding *non-agentive* aspects of our environment and subsumes the concept of ‘folk physics’. Systemizing is an *algorithmic* process: understanding systems in a relatively finite and closed fashion.

Prior work has measured individual differences in empathizing (E) and systemizing (S) with self-report questionnaires (e.g. the Empathy Quotient (EQ) (Baron-Cohen and Wheelwright, 2004) and revised Systemizing Quotient (SQ-R) (Wheelwright et al., 2006)). In the adult general population there is a significant but small inverse correlation (*r* = − 0.09) between E and S (Wheelwright et al., 2006). Furthermore, E and S co-vary mainly along their differences (i.e., in terms of their *discrepancy*) rather than their summation (Goldenfeld et al., 2005). This *E–S discrepancy*, rather than E or S alone, describes an individual's dispositional *cognitive style*, and differentiates typical males, females and people with autism spectrum conditions (ASC) (Baron-Cohen, 2003; Baron-Cohen et al., 2005). It also correlates with choice of university degree in young adults, independent of their sex (Billington et al., 2007). Those with a cognitive style of ‘Type S’ (i.e., an *S > E* profile, a stronger drive to systemize than to empathize) are more likely to choose STEM (science, technology, engineering or mathematics) subjects, whereas those with a cognitive style of ‘Type E’ (i.e., an *E > S* profile, a stronger drive to empathize than to systemize) are more likely to choose humanities.

While more is known about the E–S discrepancy at the psychological level, little is known about the underlying neurobiological basis of these individual differences. Only one functional imaging study to date has explored the E–S discrepancy. Individuals with an E > S cognitive style activate frontal operculum/inferior frontal gyrus, inferior parietal lobule and posterior temporal regions during mentalizing judgments more than those with an S > E profile, who activate parahippocampal, anterior temporal and superior frontal regions for mentalizing (Focquaert et al., 2010). In terms of structural brain indices, diffusion imaging has shown that SQ is positively correlated in males but negatively correlated in females with white matter (WM) integrity near the left occipital cuneus. Conversely, a reversed relationship occurs for EQ and WM integrity in the left superior temporal gyrus (negative correlation in males but a positive correlation in females) (Chou et al., 2011). For gray matter (GM), EQ is positively correlated with volumes of the right pars opercularis and medial prefrontal cortex in adult males (Cheng et al., 2009). While these structural imaging studies tested E and S independently, no study to date has directly investigated the structural basis of the E–S discrepancy.

In the present study we examine the underlying neuroanatomical correlates of the E–S discrepancy using voxel-based morphometry (VBM) in a large sample of Caucasian male adults in the general population. We chose to focus on male adults because the inclusion of both sexes would introduce potential confounds related to biological sex and gendered processes (e.g., differences in parenting sons versus daughters, cultural stereotypes associated with males and females, etc.). We therefore decided to investigate males only as a clear starting point.

We predicted that a stronger drive to systemize than to empathize (S > E) would be associated with brain regions related to top-down analytic reasoning (Baron-Cohen and Belmonte, 2005) subserved mainly by dorsolateral, orbital prefrontal and frontal polar cortices (Clark et al., 2010; Duncan, 2010a) or networks diffusely involving occipital, parietal, temporal, frontal, basal ganglia, and cerebellar regions that underlie deductive reasoning (Goel, 2007). We also predicted that regions supporting probabilistic inference and error monitoring such as midline frontal and cingulate regions (Behrens et al., 2007; Botvinick et al., 1999; Polli et al., 2005; Rushworth and Behrens, 2008; Taylor et al., 2006) might correlate with an S > E cognitive style. In addition, we predicted that S > E would be related to bottom-up systems subserving sensory/perceptual processing (Baron-Cohen et al., 2009) (e.g. occipital regions or parietal association areas).

In contrast, we predicted that the opposite profile (a stronger drive to empathize than to systemize, or E > S) would be reflected in variation in the so-called ‘social brain’ regions known to be involved in empathy, mentalizing, social cognition, embodiment and emotion (e.g., medial prefrontal cortex, posterior cingulate cortex, precuneus, temporo-parietal junction, superior temporal sulcus, amygdala, anterior insula, caudal anterior/middle cingulate cortex, somatosensory cortex, inferior parietal lobule and frontal operculum) (Adolphs, 2009; Amodio and Frith, 2006; Baron-Cohen et al., 1999; Chakrabarti et al., 2006; Frith, 2007; Gazzola and Keysers, 2009; Keysers et al., 2004; Lombardo et al., 2010a, 2010b, 2011; Ochsner et al., 2005; Saxe and Kanwisher, 2003; Singer et al., 2004; Wicker et al., 2003).

## Material and methods

### Participants

Eighty-eight right-handed Caucasian male adults aged 18–45 years were recruited by advertisement as control participants as part of a larger multicenter imaging study within the UK Medical Research Council (MRC) Autism Imaging Multicentre Study (AIMS) Consortium (Ecker et al., 2012). Data were collected from three centers: the Institute of Psychiatry, King's College London (N = 39); the Autism Research Centre, University of Cambridge (N = 31); and the Autism Research Group, University of Oxford (N = 18). Exclusion criteria for all participants included a history of major psychiatric disorders, head injury, genetic disorders, medical conditions affecting brain structure and function (e.g. epilepsy), a diagnosis or family history of an ASC, and use of antipsychotic medications, antidepressants, mood stabilizers or benzodiazepines. All participants gave informed written consent in accordance with the ethics approval from the National Research Ethics Committee, Suffolk, UK. Intelligence was assessed with the Wechsler Abbreviated Scale of Intelligence (WASI) (Wechsler, 1999) and all 88 participants had at least average intelligence (full-scale IQ > 85).

### E–S discrepancy

Empathizing and systemizing were measured respectively by the Empathy Quotient (EQ) (Baron-Cohen and Wheelwright, 2004) and the revised Systemizing Quotient (SQ-R) (Wheelwright et al., 2006). The EQ is a 40-item questionnaire measuring both the affective and cognitive aspects of empathy. The SQ-R is a 75-item questionnaire measuring the cognitive and behavioral features of systemizing, the drive to analyze, understand, predict, control and construct rule-based systems. Participants rated on a 4-point Likert scale (ranging from ‘strongly agree’ to ‘strongly disagree’) the extent to which each item described him. The maximum score is 80 for the EQ and 150 for the SQ-R, and the minimum is zero on both.

E–S discrepancy (i.e., ‘*D* score’; see Baron-Cohen et al., 2005; Goldenfeld et al., 2005) was quantified as the difference between standardized SQ-R and EQ scores. The raw SQ-R and EQ scores were standardized by subtracting the population mean from the score then dividing it by the maximum possible score: S = (SQ-R – <SQ-R>) / 150 and E = (EQ – <EQ>) / 80, where <SQ-R> and <EQ> were the estimated population means (55.6 for SQ-R, and 44.3 for EQ) derived from a previous large-scale study (N = 1761) of participants from the same population (i.e., the United Kingdom) (Wheelwright et al., 2006). The discrepancy between systemizing and empathizing was then quantified as *D* = (S − E) / 2. Larger *D* scores are indicative of greater drive to systemize than to empathize (i.e., S>E, either ‘Type S’ or ‘Extreme Type S’), while smaller *D* scores indicate greater drive to empathize than to systemize (i.e., E>S, either ‘Type E’ or ‘Extreme Type E’). *D* scores close to zero represent an equal drive to systemize and empathize (i.e., ‘Type B’) (Baron-Cohen et al., 2005).

**Supplementary Fig. S1:**
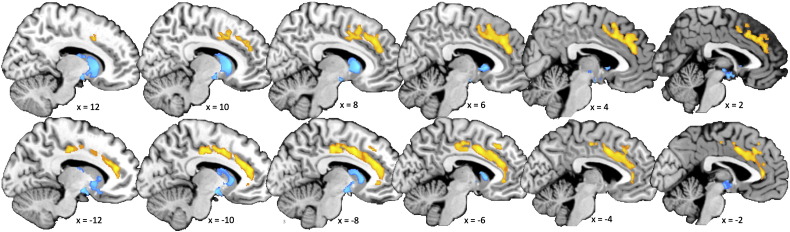
*Gray matter correlates of E–S discrepancy expressed by the D_Z_ score*. Clusters showing a volumetric correlation with the *D*_*Z*_ score (orange for S > E, blue for E > S) were overlaid on a high-resolution anatomical brain image. They were visualized according to the same thresholding criteria for statistical inferences in statistical parametric mapping (SPM) described in the Material and methods section. The results are almost exactly the same as those for the *D* score.

The E–S discrepancy was also quantified following general psychometric convention as the difference between the z-scores of the raw SQ-R and EQ scores: *D*_*Z*_ = (Z_SQ-R_ − Z_EQ_) / 2 (Wakabayashi et al., 2007). The distribution of this *D*_*Z*_ score was very similar to that of the *D* score and produced similar findings (see Supplementary Fig. S1). Therefore, results from the *D* score are presented here to be consistent with the majority of previous studies (Auyeung et al., 2009; Baron-Cohen, 2003; Baron-Cohen et al., 2005; Goldenfeld et al., 2005; Wheelwright et al., 2006).

### Structural MRI data acquisition and processing

All participants were scanned using contemporary 3T MRI scanners fitted with an 8-channel receive-only RT head-coil: GE Medical Systems HDx, Department of Radiology, University of Cambridge; GE Medical Systems HDx, Centre for Neuroimaging Sciences, Institute of Psychiatry, King's College London; Siemens Medical Systems Tim Trio, FMRIB Centre, University of Oxford. A specialized acquisition protocol employing quantitative imaging – Driven Equilibrium Single Pulse Observation of T_1_ (DESPOT1) – was utilized to ensure standardization of structural MRI scans across the three scanner platforms. This protocol has previously been validated and extensively described elsewhere (Deoni et al., 2008). In short, spoiled gradient recalled (SPGR) were acquired at two flip angles (α) from which an estimate of the absolute T_1_ value was derived at each voxel. These quantitative T_1_ maps were then used to create simulated structural T_1_-weighted inversion recovery (IR) images, with 176 contiguous slices (1 mm × 1 mm × 1 mm resolution), a field-of-view of 25.6 cm, a simulated repetition time/inversion time (TR/TI) of 1800/850 ms, a scaling constant *ρ* = 10,000 and a flip angle of 20°. This combination of parameters gave excellent deep and cortical gray/white matter contrast in the subsequent tissue segmentation without the need of modulation by B_0_ and B_1_ field inhomogeneities because compensation had been introduced during the estimation of absolute T_1_. This quantitative imaging method (Deoni, 2007) has advantages over conventional qualitative T_1_-weighted imaging because it minimizes inter-scanner/site variance in MRI measurements and improves signal-to-noise contrast.

Simulated T_1_-weighted IR images were segmented and normalized to Montreal Neurological Institute (MNI) space using SPM8 (Wellcome Department of Imaging Neuroscience Group, London, UK; http://www.fil.ion.ucl.ac.uk/spm). Native-space GM, WM and cerebrospinal fluid (CSF) images were obtained using standard automated segmentation routines. Total GM, WM and CSF volumes were estimated by summing up the partial volume estimate throughout each class of segmented images, and total brain volume (TBV) was estimated by summing up GM and WM volumes. The native-space GM and WM images were registered to a study-specific template generated from all participants using a high-dimensional non-linear diffeomorphic registration algorithm (DARTEL) (Ashburner, 2007). This non-linear warping technique minimizes inter-individual structural variance and thereby improves the sensitivity of VBM analysis. A modulation step was included to retain voxel-wise information about local tissue volume. After mapping to standard space the resulting modulated GM and WM maps were smoothed with a 4 mm full-width-half-maximum Gaussian kernel. A 4 mm smoothing kernel was chosen to retain finer local information because DARTEL provides more accurate registration than conventional normalization methods, and because 4 mm smoothing has been shown to give adequate specificity when processing through DARTEL (Henley et al., 2010).

### Statistical analysis

Voxel-wise statistical testing on the modulated GM and WM images, respectively, was performed with SPM8. To avoid possible edge effects between different tissue types, all voxels with a value (i.e., partial volume estimate) less than 0.25 were excluded from analysis. A general linear model was then regressed at each voxel where the *D* score was the independent variable of interest, with centers (scanning machines) as categorical fixed-effect factors, and TBV and age as covariates. Including between-center components of variance in the statistical model has been proved necessary and adequate for multi-center imaging studies (Suckling et al., 2010, 2012). Both positive and negative correlations with *D* score were corrected for multiple comparisons by controlling the topological false discovery rate (FDR) at *q* < 0.05 for clusters, using a cluster-forming height threshold of *p* < 0.025 for each contrast and a spatial extent threshold (FDRc) calculated under Gaussian Random Field Theory (Chumbley and Friston, 2009). This extent threshold was also corrected for non-stationarity (Hayasaka et al., 2004). The same procedures were performed again with the *D*_*Z*_ score, EQ or SQ-R scores respectively being the independent variables of interest within the model (see supplementary results and figures).

## Results

### E-S discrepancy

**Fig. 1:**
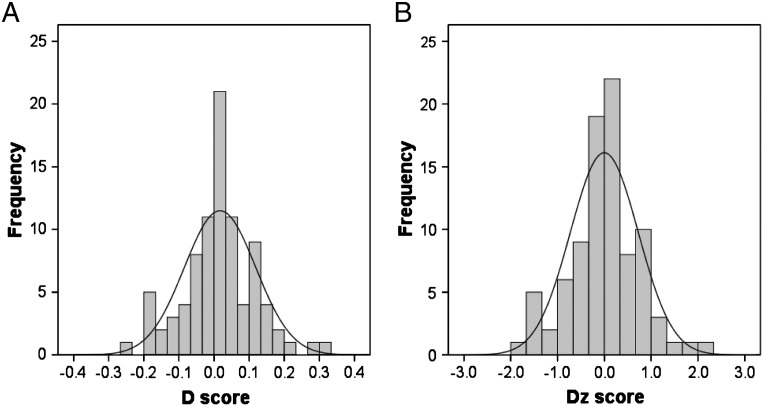
*The distribution of D and D*_*Z*_*scores*. E–S discrepancy was quantified as the difference between standardized measures of systemizing and empathizing. Panels A and B illustrate the distribution of the D and D_Z_ scores, respectively. They were generated from the same raw scores with slightly different standardization strategies; see Material and methods for detail. Both scores, representing dispositional cognitive style, were distributed with large variability and were not significantly deviant from normality.

The distributions of SQ-R and EQ scores did not deviate from normality (*p* = 0.88 for SQ-R and 0.15 for EQ, Shapiro–Wilk normality test) and the means were comparable to the estimated population means from the previous large-scale study of the SQ-R (55.6) and EQ (44.3) (Wheelwright et al., 2006); see Table 1. SQ-R and EQ scores in this sample were not significantly correlated (*r* = − 0.05, *p* = 0.62). The distribution of *D* did not deviate from normality (*p* = 0.34, Shapiro–Wilk normality test), with a skewness of 0.115 (standard error, SE = 0.257) and a kurtosis of 0.616 (SE = 0.508). The distribution of *D*_*Z*_ was similar to *D*; see Fig. 1. Percentages of the sample belonging to each categorical cognitive style defined in previous literature (Wheelwright et al., 2006) are provided in Supplementary Table S1. There were no significant correlations between *D* and age (*r* = 0.031, *p* = 0.77), verbal IQ (*r* = − 0.007, *p* = 0.95), performance IQ (*r* = − 0.005, *p* = 0.97) or full-scale IQ (*r* = 0.002, *p* = 0.99).

Sixty-three of the 88 participants reported their higher education degree subjects. There was no difference in *D* between those with and without degree subject information. Among the 63 participants who reported their degree, 23 studied humanities, 12 studied psychology or social sciences, and 28 studied physical/natural sciences; categorization of degree subjects followed our previous study (Billington et al., 2007). To test if findings from Billington et al. replicated, we compared *D* between those who studied physical/natural sciences and humanities, and predicted a higher *D* of the former than the latter. This prediction was confirmed (physical/natural sciences: mean *D* = 0.038, standard deviation, SD = 0.117; humanities: mean *D* = − 0.015, SD = 0.072; one-tailed *p* = 0.031). This further validates *D* (and its neural basis) as having a meaningful behavioral correlate in the real world.

### Brain structural correlates to *D* score

**Supplementary Fig. S2:**
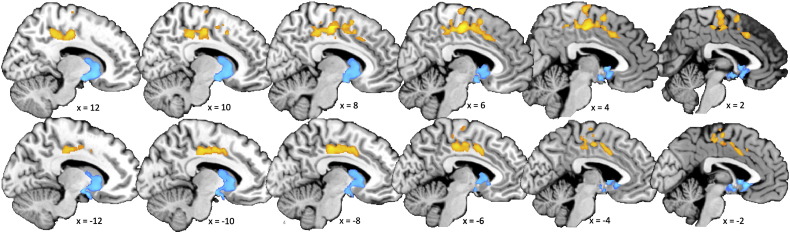
*Gray matter correlates of SQ-R*. Clusters showing a volumetric correlation with the SQ-R score (orange for positive correlation, blue for negative correlation) were overlaid on a high-resolution anatomical brain image. They were visualized according to the same thresholding criteria for statistical inferences in SPM described in the Material and methods section.

Because analyses using *D* and *D*_*Z*_ produced similar results, we will continue using results from the analysis on *D* rather than *D*_*Z*_, to be consistent with the majority of previous studies leading up to this work (Auyeung et al., 2009; Baron-Cohen, 2003; Baron-Cohen et al., 2005; Goldenfeld et al., 2005; Wheelwright et al., 2006). See Supplementary Fig. S1 for results from the *D*_*Z*_ score. Also see Supplementary Results and Supplementary (Fig. S2) for analyses on just EQ or SQ-R scores alone.

**Fig. 2:**
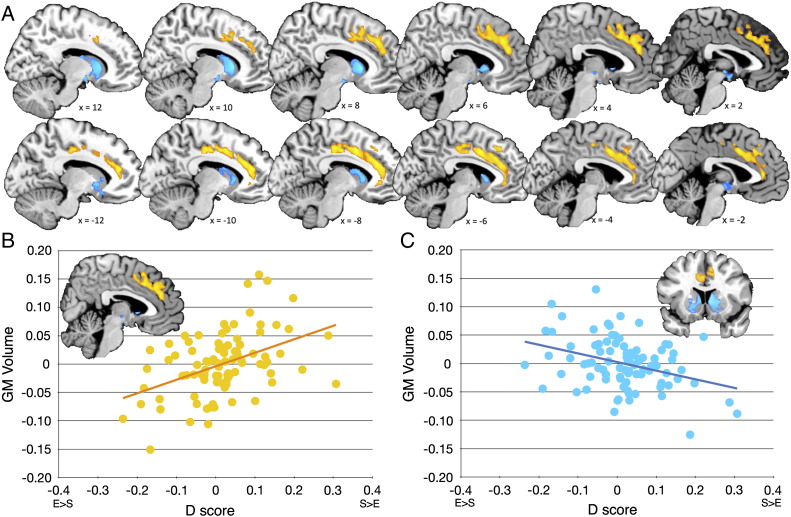
*Gray matter correlates of E–S discrepancy*. Clusters showing a volumetric correlation with the *D* score were overlaid on a high-resolution anatomical brain image. They were visualized according to the same thresholding criteria for statistical inferences in statistical parametric mapping (SPM) described in the Material and methods section. Panel A illustrates bilateral midline prefrontal and anterior/middle cingulate structures (marked in orange) whose size was positively correlated with *D*. Here, a stronger drive to systemize than to empathize was associated with a larger relative regional volume; see panel B. Panel A also illustrates hypothalamus and bilateral ventral basal ganglia (marked in blue) whose size was negatively correlated with *D*. Here, a stronger drive to empathize than to systemize was associated with a larger relative regional volume; see panel C. In panels B and C, the x-axis represents the *D* score and the y-axis indicates the residual GM volume of the clusters (i.e., after regressing out centers, total brain volume and age effects). The scatter plots are presented here solely for illustrating the distribution of the data. The size and nature of the correlation displayed should not be used for inference and interpretation on the effect size, as SPM has been used to make the primary inferences.

For GM, we observed one cluster (cluster size k_e_ = 8272 voxels, cluster-level *p* < 0.001, peak-voxel [MNI coordinate: − 6, 8, 39] T = 3.96) that was significantly *positively* correlated with *D*. This meant that a stronger drive to systemize than to empathize was related to larger relative GM volume. This cluster extended across bilateral anterior cortical midline structures including the anterior and middle cingulate cortices (ACC/MCC), paracingulate cortices and dorsal medial prefrontal cortices (dMPFC); see orange cluster in Fig. 2A.

In the other direction, we observed two clusters that were *negatively* correlated with *D*. This meant that a stronger drive to empathize than to systemize was related to larger relative GM volume. These two clusters (right-sided cluster: k_e_ = 5736 voxels, cluster-level *p* = 0.007, peak-voxel [6, 16, 3] T = 3.69; left-sided cluster: k_e_ = 3981 voxels, cluster-level *p* = 0.021, peak-voxel [− 26, − 3, − 0] T = 3.36) were located in hypothalamus and bilateral ventral basal ganglia (i.e., nucleus accumbens and ventral caudate/putamen, and parts of pallidum); see blue clusters in Fig. 2A.

For WM, no regions were related to *D* after whole-brain correction for multiple comparisons.

## Discussion

This is the first study to identify the neuroanatomical basis of the discrepancy between systemizing and empathizing in adult males. This *E–S discrepancy* (or *D*) reflects a dimension of individual differences in dispositional cognitive style, which is normally distributed and independent of age and IQ. It also relates to choice of certain higher education degree subjects (humanities versus physical/natural sciences) in this sample, replicating a previous report (Billington et al., 2007). We identified several regions where local relative GM volume was related to increasing S > E or E > S profiles. A stronger drive to systemize than to empathize (S > E) was related to larger volume of bilateral midline cingulate, paracingulate and prefrontal structures, whereas a stronger drive to empathize than to systemize (E > S) was related to larger subcortical regions including the hypothalamus and bilateral ventral basal ganglia (particularly ventral striatum). No WM region was found to have volumetric correlation to E–S discrepancy.

**Fig. 3:**
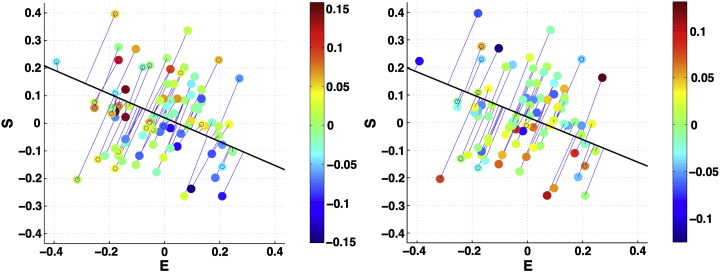
*Joint contribution of E and S to the D–GM relationship.* These two-dimensional scatterplots illustrate projections onto the E–S plane of the three-dimensional E, S and GM volume scatterplots. Left: positive correlation between *D* and GM volume in ACC/MCC/paracingulate/dMPFC (Supplementary Movie S1); Right: negative correlation between *D* and GM volume in ventral basal ganglia/hypothalamus (Supplementary Movie S2). Datapoints are color coded to represent magnitude of residual GM volume (i.e., after regressing out centers, total brain volume and age effects) and the solid line is the E–S plane projection of a linear least-squares regression fitted to the three-dimensional data, which represents the first principal component of this relationship, and indicates its E–S component. Viewing the data in this way allows visualization of the relative contributions of E and S to the *D*–GM volume relationship. If S was the sole contributor and E made no contribution, the vector would be parallel to the S axis, and vice versa. The diagonal slope indicates that both E and S make joint contributions to the overall *D*–GM volume relationship.

*D* is operationalized as the standardized difference (i.e., discrepancy) between S and E, hence a dimensionality reduction. Although *D* is a parsimonious and meaningful reflection of cognitive styles: how do S and E contribute to the *D*–GM volume correlation? Deconstructing *D* to its component variables, S and E, reveals an underlying relationship among E, S and GM volume; see Supplementary Movies S1 and S2 and Fig. 3. The observed *D*–GM volume relationship reflects the joint contribution of both E and S rather than either E or S being the primary contributor; see Fig. 3. Thus, it is the combined effect of E and S on *D* that is important in understanding why *D*, and not E or S alone, best describes dispositional cognitive styles and predicts degree choice.

The functions of regions related to the E–S discrepancy may provide important insights into the component processes that define such cognitive styles. The cortical midline cluster associated with greater S > E is important in the detection, prediction and monitoring of errors (Botvinick et al., 1999; Polli et al., 2005; Rushworth and Behrens, 2008; Taylor et al., 2006). ACC/MCC also has an important role in tracking higher-order probabilistic statistics of the environment such as volatility (Behrens et al., 2007). Even in social contexts, areas such as dMPFC are important for tracking prediction errors and volatility of social information (Behrens et al., 2008), the drive to acquire more social information (Rudebeck et al., 2006), strategic social reasoning (Coricelli and Nagel, 2009), degree of sophistication in belief inferences (Yoshida et al., 2010), and higher-order computations of how one influences others (Hampton et al., 2008). Since an individual high in S > E should have a strong preference for identifying probabilistic regularities that govern how a system (social or nonsocial) behaves (Baron-Cohen, 2006), these functions are compatible with what would be predicted for an S > E dispositional cognitive style. Though it is not ideal to make reverse inferences about the processes important in an S > E cognitive style based solely on the findings summarized above, they suggest directions for future work into the component cognitive processes that make up an S > E profile (and systemizing itself). For example, this work would predict that processes such as estimation of volatility in both social and nonsocial environments would be integral for an individual high in S > E. Tasks designed to probe these and other functions of the midline regions may provide insight into the mechanisms underlying the E–S discrepancy, and may suggest new tests for individuals with ASC, where the E–S discrepancy is exaggerated (Baron-Cohen et al., 2005).

We did not find any relationship between S > E and brain structures related to sensory/perceptual processing (e.g. occipital and parietal association areas). One reason for this lack of a relationship may be because items on the SQ-R mainly measure the analytic, monitoring and prediction processes rather than the sensory/perceptual aspects. Similarly, one reason why lateral prefrontal structures and many other regions involved in deductive reasoning (Goel, 2007) were not related to S > E may be because the questionnaires measure dispositional traits and drives, rather than specific cognitive abilities underlying executive functions and intelligence (Duncan, 2010a) supported by these regions. The lack of a correlation between *D* and IQ supports this dissociation between disposition and ability.

Surprisingly, the predicted relationship between E > S and volume of ‘social brain’ regions (Adolphs, 2009; Frith, 2007) was not observed. Instead, we found a robust association between E > S and volume of the ventral basal ganglia (particularly the ventral striatum) and hypothalamus. The ventral striatum is a core structure of the dopaminergic system, integral in modulating motivation and learning (Bjorklund and Dunnett, 2007; Graybiel, 1995; Krack et al., 2010) through both non-social and social rewards (Haber and Knutson, 2010). It processes primary rewards (e.g. food and sexual stimuli) (Arnow et al., 2002; Berns et al., 2001; McClure et al., 2003; Pagnoni et al., 2002), secondary rewards (e.g. money) (Breiter et al., 2001; Delgado et al., 2000), and even emotional rewards related to humor (Mobbs et al., 2003). It also processes social rewards in a similar manner to monetary rewards (Izuma et al., 2008), vicarious rewards (Mobbs et al., 2009), and the sense of being liked (Davey et al., 2010). In addition, the personality trait of ‘reward dependence’ measured by the Cloninger's temperament and character inventory (Cloninger, 1994) is positively correlated with gray matter density of the ventral striatum (Lebreton et al., 2009). On the other hand, the hypothalamus plays a critical role in emotion, motivation and behavior, as well as in controlling and modulating the endocrine system. Perhaps the most relevant neuroendocrine influences for empathizing and social behavior are oxytocin and testosterone. Both hormones play a role in modulating sensitivity and responsiveness to emotion, social cues and rewards (Bartz et al., 2011; Bos et al., 2012; Hermans et al., 2010; van Honk et al., 2011). In sum, this unexpected yet novel finding suggests that one of the main differences between the brains of individuals that vary in E–S discrepancy is variation in systems involved in reward processing and neuroendocrine control. Future studies should investigate such systems in ASC, where the E–S discrepancy is substantial. Taken together with the S > E results, these findings may suggest that one primary difference between S > E and E > S individuals is ‘modes’ of learning. It may be that E > S individuals are more affected by (socially mediated) reward-dependent learning, while S > E individuals are more affected by (non-socially mediated) probabilistic learning.

Another facet of the results that is important to address is why E > S was not associated with the size of ‘social brain’ regions. We know that EQ correlates highly with the agreeableness dimension of the Big-Five personality construct (Nettle, 2007), and agreeableness correlates to volume of certain components of the ‘social brain’ (i.e., superior temporal sulcus, posterior cingulate, fusiform gyrus) (DeYoung et al., 2010). However, E > S profile is not a total reflection of the broad construct of empathy, but a stronger drive to empathize than to systemize. Therefore, the inclusion of variability related to S in a measure like *D* may change such predicted relationships with areas typically viewed as related to various aspects of social cognition. Empathy encompasses both controlled/analytical/algorithmic and automatic/intuitive/heuristic processes. E > S is a profile in individuals whose cognitive style is more suited to open systems (i.e., automatic, intuitive, and heuristic-based), while S > E is a profile in individuals whose cognitive style is more suited to assessing closed systems (i.e., controlled, analytical, and algorithmic). The present results therefore indicate that the underlying component processes behind an E > S cognitive style (as one aspect of the broad construct of empathy) are not just those related to functions subserved by brain regions typically associated with social cognition. Rather, they suggest a new avenue for studying empathy and E > S style via links between reward processing and automatic/intuitive/heuristic styles of information processing.

It is worth noting that a well known ‘theory of mind’ region in the social brain, dMPFC (Amodio and Frith, 2006), was correlated with S > E and not E > S. This result may seem difficult at a first glance. However, it may have more to do with prior notions about the function of dMPFC than anything else. Although dMPFC is known to be reliably involved in mentalizing and explicit social cognitive processing, recent work shows that its involvement in social cognition may be more tuned to an individual high in S > E. Several studies using computational modeling have shown that dMPFC is increasingly involved in social cognitive processing as a function of the complexity of the processing (Coricelli and Nagel, 2009; Hampton et al., 2008; Yoshida et al., 2010). Other work has suggested that dMPFC function is not specific to social cognition but is also involved in intelligence (Shaw et al., 2006) and other higher-order non-social cognitive processes such as tasks with multiple demands (Duncan, 2010b; Roca et al., 2011). As we understand more about the function of dMPFC in higher-order cognitive processing we may find that simplistic categorization as ‘social’ and ‘non-social’ may not fit. For interpreting the current set of results, we suggest that one reason for finding a relationship between dMPFC and S > E rather than E > S may have to do with dMPFC's role in cognitive processing at increasingly higher-orders of complexity.

There are some limitations of the current study. First, VBM is a mass-univariate statistical approach. Though spatially unbiased and with large exploratory power in detecting volumetric differences, it is not suitable for delineating multivariate features of the brain (e.g. its shape), and it cannot disentangle the complex geometric features that contribute to cortical volume such as surface area and cortical thickness. While this is not an issue for interpreting the subcortical results, the cortical midline findings may be colored by contributions from either. Second, although a larger GM volume in typical adults is usually interpreted as reflecting an enhanced function (Fleming et al., 2010; Kanai and Rees, 2011; Maguire et al., 2000), it is not the only plausible interpretation. It is still unclear what a greater GM volume reflects at the cellular level (Kanai and Rees, 2011; Terrazas and McNaughton, 2000) (e.g., increased number of neurons or glia cells, more complex axonal or dendritic connections, higher or lower synaptic density, etc.). The interpretation of what a greater GM volume means in relation to cognition depends on what it reflects at the cellular and physiological levels. For instance, some studies have shown that GM volume is negatively correlated with cognitive performance in adolescents (Dumontheil et al., 2010), which may be explained developmentally by synaptic pruning (Kanai and Rees, 2011). Thus, further work is needed to fully tease apart these relationships and what they may mean in terms of cognitive function.

Third, the E–S discrepancy is currently defined by self-report, and how this corresponds to experimental performance measures is not fully established. In addition, the current findings are correlational so any causal inferences between cognitive style and brain structure require further testing. Finally, the present results are only from male adults. Although this presents a picture free from potential confounds of biological sex and gendered processes, whether females have comparable neuroanatomical correlates awaits future investigation. The same applies to children, adolescents and older populations. Our ongoing studies will extend the current approach to females and other age groups. Despite these limitations, this work suggests future directions to illuminate the neuropsychological and functional correlates of cognitive styles.

In conclusion, for male adults, the discrepancy between empathizing and systemizing is related to distinct individual differences in brain structure. Men with a stronger drive to systemize than to empathize have increasingly larger midline cingulate and prefrontal structures, whereas those with a stronger drive to empathize than to systemize have an increasingly larger ventral basal ganglia and hypothalamus. These findings provide insights into the biological basis of individual differences in empathizing and systemizing, and point to directions for future studies into the neuropsychological basis of dispositional cognitive styles.

The following are the supplementary materials related to this article.

Supplementary materials.

Supplementary Movie S1 *Three-dimensional scatterplots with total least squares fitted line of the relationship between E, S and GM volume.* Movie S1 shows a three-dimensional scatterplot of standardized E and S scores and residual GM volume (i.e., after regressing out centers, total brain volume and age effects) of the cortical midline cluster at medial prefrontal cortex (MPFC, including ACC, MCC, paracingulate, and dMPFC). Coloring of datapoints represents magnitude on z-axis (i.e., residual GM volume).

Supplementary Movie S2 *Three-dimensional scatterplots with total least squares fitted line of the relationship between E, S and GM volume.* Movie S2 shows a three-dimensional scatterplot of standardized E and S scores and residual GM volume of the subcortical clusters at ventral basal ganglia and hypothalamus. Coloring of datapoints represents magnitude on z-axis (i.e., residual GM volume).

## Figures and Tables

**Table 1:** Participant characteristics.

*N = 88*	Mean	SD	Range	Comparison with Wheelwright et al. (2006); $
Age (in years)	29.0	7.1	18–45	–
VIQ	110.4	12.6	71–141	–
PIQ	116.8	10.6	93–135	–
FIQ	115.0	11.3	86–137	–
SQ-R	59.2	20.3	16–115	*t*_(87)_ = 1.68 (95% CI = − 0.67–7.95), *p* = 0.10
EQ	43.5	11.6	13–66	*t*_(87)_ = − 0.64 (95% CI = − 3.24–1.66), *p* = 0.52
*D* score	0.017	0.102	− 0.236–0.307	–

SD = standard deviation; VIQ = verbal IQ; PIQ = performance IQ; FIQ = full-scale IQ; CI = confidence interval.

$ : One-sample *t* test.
