# Supplementary material for: Individual differences in brain structure underpin empathizing–systemizing cognitive styles in male adults
Source: Neuroimage. 2012 Jul 16;61(4):1347–54. doi: 10.1016/j.neuroimage.2012.03.018 (PMC3381228; doi:10.1016/j.neuroimage.2012.03.018)
Supplement: Supplementary file 1 — Supplementary materials. [file mmc1.doc]

*Supplementary Results: GM correlates of EQ and SQ-R scores*

Using the same statistical modeling and thresholding procedures, we additionally examined the GM correlates of EQ and SQ-R scores separately. There were no regions where relative GM volume significantly correlated with EQ score after whole-brain correction for multiple comparisons. However for SQ-R, we observed one cluster located bilaterally at the posterior and middle cingulate cortices (PCC/MCC) and supplementary motor area (SMA) (cluster size k_e_ = 6,811 voxels, cluster-level *p* < 0.001, peak-voxel [MNI coordinate: 8, -15, 53] T = 5.24) showing a significant positive correlation with GM volume; meanwhile, we observed one cluster located at the bilateral ventral basal ganglia and hypothalamus (cluster size k_e_ = 15,587 voxels, cluster-level *p* < 0.001, peak-voxel [MNI coordinate: 23, 0, -4] T = 4.46) showing a significant negative correlation with GM volume; see Supplementary Figure S2. The latter cluster was at nearly identical regions showing a negative correlation to *D*, as described in the main text.

It is important to note that the null finding for EQ and the nearly identical regions showing negative correlations to both *D* and SQ-R together should *not* be misinterpreted as suggesting that the neuroanatomical correlates of individual differences in *D* were driven mainly by individual differences in systemizing. Finding similar brain correlates between *D* and S (SQ-R) but not E (EQ) does not imply that S contributes more to the variance of *D* than E does; these are basically two separate analyses (see Nieuwenhuis et al. for a relevant ‘alarm’). Instead, contributions of E and S to *D* are appropriately addressed by decomposing *D* back to E and S within the context of the correlation analysis between *D* and brain volumes; see Figure 3 and Supplementary Movies S1 and S2.

*Supplementary Table S1. Percentages for each categorical cognitive style (defined by Wheelwright et al. )*

| Cognitive style | Boundary | Percentage (%) |
| --- | --- | --- |
| Extreme Type E | *D* < -0.21 | 1.1 |
| Type E | -0.21 ≤ *D* < -0.041 | 23.9 |
| Type B | -0.041 ≤ *D* < 0.040 | 38.6 |
| Type S | 0.040 ≤ *D* < 0.21 | 33.0 |
| Extreme Type S | *D* ≥ 0.21 | 3.4 |

*Supplementary Figure S1. Gray matter correlates of E-S discrepancy expressed by the D_Z_ score*

Clusters showing a volumetric correlation with the *D_Z_* score (orange for S>E, blue for E>S) were overlaid on a high-resolution anatomical brain image. They were visualized according to the same thresholding criteria for statistical inferences in statistical parametric mapping (SPM) described in the Material and Methods section. The results are almost exactly the same as those for the *D* score.

*Supplementary Figure S2. Gray matter correlates of SQ-R*

Clusters showing a volumetric correlation with the SQ-R score (orange for positive correlation, blue for negative correlation) were overlaid on a high-resolution anatomical brain image. They were visualized according to the same thresholding criteria for statistical inferences in SPM described in the Material and Methods section.

*Supplementary Movies S1 and S2: Three-dimensional scatterplots with Total Least Squares fitted line of the relationship between E, S and GM volume*

Movie S1 shows a three-dimensional scatterplot of standardized E and S scores and residual GM volume (i.e., after regressing out centers, total brain volume and age effects) of the cortical midline cluster at medial prefrontal cortex (MPFC, including ACC/MCC/paracingulate/dMPFC). Movie S2 shows a three-dimensional scatterplot of standardized E and S scores and residual GM volume of the subcortical clusters at ventral basal ganglia/hypothalamus. Coloring of datapoints represents magnitude on z-axis (i.e., residual GM volume).

**Reference**

Nieuwenhuis, S., Forstmann, B.U., Wagenmakers, E.J., 2011. Erroneous analyses of interactions in neuroscience: a problem of significance. Nat Neurosci 14, 1105-1107.

Wheelwright, S., Baron-Cohen, S., Goldenfeld, N., Delaney, J., Fine, D., Smith, R., Weil, L., Wakabayashi, A., 2006. Predicting Autism Spectrum Quotient (AQ) from the Systemizing Quotient-Revised (SQ-R) and Empathy Quotient (EQ). Brain Res 1079, 47-56.
