# Supplementary figures and images for: Individual differences in brain structure underpin empathizing–systemizing cognitive styles in male adults
Source: Neuroimage. 2012 Jul 16;61(4):1347–54. doi: 10.1016/j.neuroimage.2012.03.018 (PMC3381228; doi:10.1016/j.neuroimage.2012.03.018)

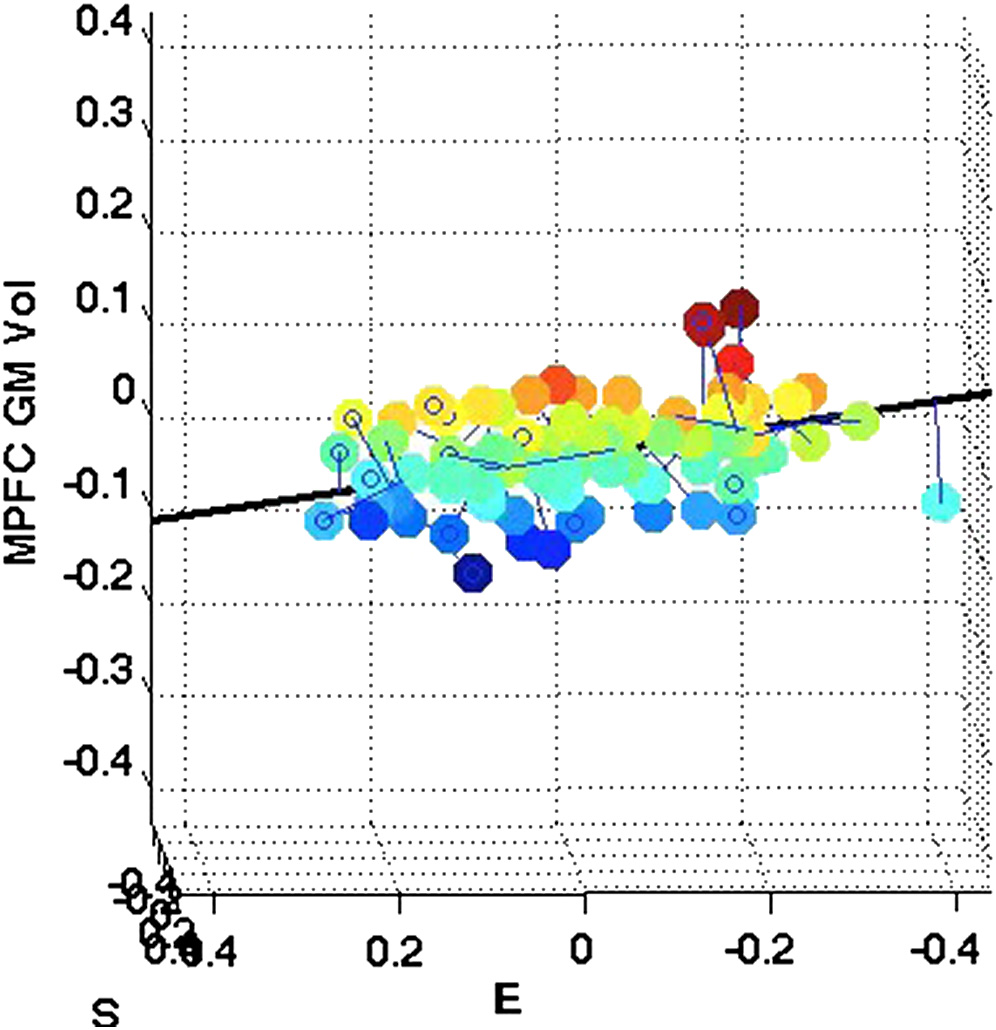

Supplement: Supplementary Movie S1 — Three-dimensional scatterplots with total least squares fitted line of the relationship between E, S and GM volume. Movie S1 shows a three-dimensional scatterplot of standardized E and S scores and residual GM volume (i.e., after regressing out centers, total brain volume and age effects) of the cortical midline cluster at medial prefrontal cortex (MPFC, including ACC, MCC, paracingulate, and dMPFC). Coloring of datapoints represents magnitude on z-axis (i.e., residual GM volume). [file mmc2.jpg]

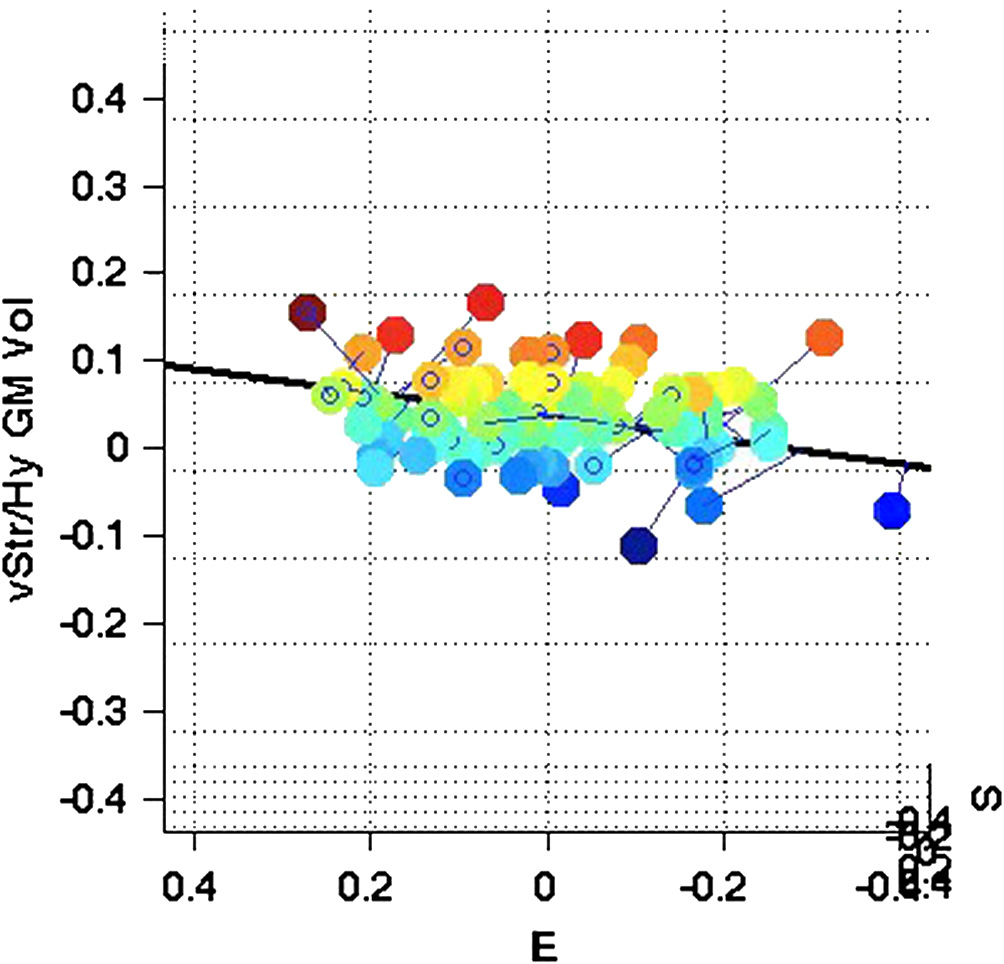

Supplement: Supplementary Movie S2 — Three-dimensional scatterplots with total least squares fitted line of the relationship between E, S and GM volume. Movie S2 shows a three-dimensional scatterplot of standardized E and S scores and residual GM volume of the subcortical clusters at ventral basal ganglia and hypothalamus. Coloring of datapoints represents magnitude on z-axis (i.e., residual GM volume). [file mmc3.jpg]
